# Capsule Endoscopy for Crohn's Disease: Current Status of Diagnosis and Management

**DOI:** 10.1155/2016/8236367

**Published:** 2015-12-27

**Authors:** Dong-Hoon Yang, Bora Keum, Yoon Tae Jeen

**Affiliations:** ^1^Department of Gastroenterology, Asan Medical Center, University of Ulsan College of Medicine, Seoul, Republic of Korea; ^2^Department of Internal Medicine, Korea University Anam Hospital, Korea University College of Medicine, Seoul, Republic of Korea

## Abstract

Crohn's disease (CD) is an idiopathic inflammatory bowel disease involving the small and/or large intestine. More than 50% of Western CD patients and up to 88% of Asian CD patients may have small intestinal involvement. Video capsule endoscopy (VCE) has a higher diagnostic yield than small bowel barium radiography and computed tomography enterography for the detection of small intestinal involvement of CD. VCE also provides diagnostic yields comparable to magnetic resonance- (MR-) based enterography or enteroclysis and may have several advantages over MR-based tests for the detection of early small intestinal lesions. Several studies have suggested the use of VCE-based disease activity scoring systems to evaluate small intestinal mucosal disease activity, although their clinical relevance needs to be further studied. A possible indication for VCE is recurrence monitoring after complete surgical excision of CD-involved segments but its usefulness and efficacy compared with conventional endoscopy should be evaluated. The capsule retention rate ranges from 0 to 5.4% in suspected CD patients and from 0 to 13.2% in established CD patients. If VCE is necessary, significant small bowel stricture should be ruled out before VCE by performing a patency capsule study and/or small bowel radiological study in suspected or established CD patients.

## 1. Introduction

Crohn's disease (CD) is a chronic, idiopathic inflammatory bowel disease (IBD) that mainly involves the small and/or large intestines. According to population-based epidemiologic studies, more than 50% of Western CD patients [1–5] and 77–87.7% of Asian CD patients have small bowel involvement at diagnosis [6–8]. Therefore, evaluation of the small and large intestine is essential for the diagnosis of CD, but there are no known pathognomonic features in endoscopic and radiologic studies.

Given that mucosal healing is considered a target of CD treatment in the biologic era [9], the endoscopic evaluation of the intestinal mucosa is crucial for assessing the treatment response and establishing a treatment strategy. In the past, small bowel follow-through (SBFT), enteroclysis (EC), and surgical exploration were the only methods for evaluating the status of the small intestine in CD patients. With technological advances, computed tomography- (CT-) based imaging protocols, such as CT enterography (CTE)/CT enteroclysis (CTEC), and magnetic resonance- (MR-) based imaging protocols, such as MR enterography (MRE)/MR enteroclysis (MREC), have become available for the radiological investigation of small intestinal lesions. However, none of these radiologic modalities can provide direct visualization of the small bowel mucosa and, thus, all carry an intrinsic limitation in the precise assessment of mucosal healing.

The introduction of wireless video capsule endoscopy (VCE) and device-assisted enteroscopy has enabled the direct visualization of the small intestinal mucosa. Because of the reduced invasiveness of VCE compared with device-assisted enteroscopy, VCE is more suitable for the diagnosis of CD and the assessment of the treatment response, unless the patients have suspected intestinal strictures that can potentially cause capsule retention. Our present review is focused on the clinical usefulness and application of VCE in CD.

## 2. Diagnostic Yield and Performance of VCE in CD

CD frequently involves the terminal ileum which usually can be evaluated using ileocolonoscopy. However, the involvement of small intestine more proximal to terminal ileum is common in CD and often affects the patients' clinical manifestation and outcomes. Small bowel barium radiography, including SBFT and EC, was a traditional diagnostic modality for evaluating the entire small intestine but has been progressively substituted by cross-sectional radiologic tests such as CTE/CTEC and MRE/MREC for the evaluation of the patients with suspected or established small intestinal CD. However, given that the VCE can visualize the intestinal mucosa directly, VCE is more likely to detect earlier lesions or subtle mucosal lesions than small intestinal radiologic studies including small bowel barium radiography, CTE, and MRE. Many investigators have compared the diagnostic yield of VCE with that of other radiologic studies in the patients with suspected or established CD.

Comparing with small bowel barium radiography, such as SBFT or EC, VCE showed better diagnostic yield for the patients having suspected CD (Table 1). The reported diagnostic yield of VCE ranged from 49% to 77%, whereas those of SBFT and EC ranged from 21% to 67% [10–13] and from 12% to 36% [14–16], respectively. Although the number of participants in each study was small, meta-analyses reported again that VCE is superior to small bowel barium radiography in terms of diagnostic yield [17–19].

The reported diagnostic yield of VCE was higher than that of CTE/CTEC in most previous studies (Table 2). The diagnostic yield was 61% for VCE and 29% for CTEC according to a prospective comparison of VCE and CTEC for the diagnosis of small bowel involvement in established CD patients [20]. The diagnostic yields of VCE and CTE in three studies of suspected or established CD patients ranged from 30% to 77% and from 33% to 53%, respectively [12, 21, 22]. Two of these studies reported a better diagnostic yield of VCE compared with CTE [12, 21] but the other study, using ileocolonoscopic findings and surgery as the gold standards, reported a similar diagnostic yield of the two modalities for terminal ileal CD [22]. However, although the diagnostic yield was similar between VCE and CTE in that larger study (30% for VCE and 33% for CTE), VCE showed higher sensitivity and specificity for the detection of terminal ileal lesions than CTE (sensitivity, 100% for VCE versus 76% for CTE; specificity, 91% for VCE versus 85% for CTE) [22]. On the other hand, another study reported that the sensitivity of VCE was similar to that of CTE but that the specificity was inferior to that of CTE [23]. The sensitivity and specificity of VCE and CTE/CTEC may result from different definitions of positive findings between two modalities, but this study concluded the diagnosis of small intestinal CD based on the consensus panel of the coinvestigators instead of specifying findings for diagnosis of small intestinal involvement. Considering the nature of each diagnostic modality and the limited number of participants in most studies, if the proportion of CD patients having subtle small intestinal lesions was small, such studies could not show the superiority of VCE in the diagnostic yield comparing with CTE/CTEC. Although a few studies did not show the superiority in diagnostic yield, recent meta-analyses concluded that VCE had superior diagnostic yield in the small bowel CD compared with CTE/CTEC.

Several prospective studies reported the diagnostic yield or performance of VCE and MRE/MREC (Table 3) and a recent meta-analysis that included five prospective studies reported that the effectiveness of VCE and MRE/MREC in the diagnosis of small bowel CD was comparable [18]. In spite of similar diagnostic yields between two modalities, VCE showed better sensitivity than MRE/MREC in several studies. One study suggested MREC was superior to VCE in the diagnostic performance. However, the diagnostic performance of MREC in all participants (including 34% of stenotic CD) was compared with the diagnostic performance of VCE in the nonstenotic CD [24]. Therefore, this study is not suitable to conclude the difference in the diagnostic performance between VCE and MREC for the nonstenotic CD patients.

Four prospective studies [21, 25–27] and two abstracts [28, 29] have compared the diagnostic yield of VCE and ileocolonoscopy in CD patients (Table 4). According to a recent meta-analysis that included these six studies, the weighted incremental diagnostic yield of VCE compared with ileocolonoscopy was 0.12 (95% confidence interval [CI], 0.00–0.23; *P* = 0.04). Because push enteroscopy cannot effectively shorten the bowel, the depth of insertion is usually less than 120 cm from the Treitz ligament, even with an overtube [30, 31]. VCE and push enteroscopy in small bowel CD patients have been described in only one prospective study [15] and one abstract [28]. A meta-analysis of these two studies showed that the weighted incremental diagnostic yield of VCE compared with push enteroscopy was 0.43 (95% CI, 0.32–0.53; *P* < 0.00001) [18].

## 3. Clinical Application of VCE in CD

### 3.1. Diagnosis of CD

According to the previous studies, the diagnostic yield of VCE is better than that of SBFT or CTE/CTEC and comparable to MRE/MREC. However, before performing VCE, clinician should take into account several things. First, diagnostic yield does not mean diagnostic accuracy. The diagnostic yield does not mean the number of tests with “diagnostic” findings among the total number of tests but the number of tests with abnormal findings among the total number of tests. Moreover, there are no available standardized VCE findings for diagnostic or specific to CD. Therefore, a high diagnostic yield in a certain study does not indicate high diagnostic accuracy and the diagnostic yield can also be influenced by the study participants' likelihood of having CD. In a previous study, for example, the yield of VCE for the evaluation of abdominal pain and/or diarrhea lasting longer than 6 months was low [35], but 12 of 17 patients with nondiagnostic ileocolonoscopic and radiologic studies, who had abdominal pain, diarrhea, anemia, and weight loss for 6.3 years on average, showed abnormal VCE findings compatible with CD [36]. The international conference on capsule endoscopy (ICCE) recommended that VCE should be considered for the patients with suspected CD if they present with typical symptoms plus either extraintestinal manifestations, abnormal inflammatory markers, or abnormal small bowel imaging [37], and a retrospective study showed that the 57.9% of patients fulfilling two criteria and 77.8% of patient fulfilling 3 or more criteria showed significant VCE findings but only 17.8% of patients showed significant VCE findings when they did not meet ICCE criteria [38].

Second, suspected and established CD is the major risk factor of capsule retention and thus, the presence of stricture should be assessed before performing VCE. According to a systematic review, the pooled capsule retention rate for all indications was 1.4% and the retention rate for indications of CD was 2.6%. The retention rate in suspected CD patients and established CD patients ranged from 0% to 5.4% and from 0% to 13.2%, respectively [11, 13, 14, 20, 36, 39–43]. Six prospective studies reported that VCE was successfully completed without retention in all patients with suspected intestinal stricture after passage of an intact patency capsule [44–49]. A retrospective study comparing patency capsule with radiologic studies (10 CT, 29 CTE, 9 MRE, and 2 SBFT) for the detection of significant strictures in order to avoid capsule retention reported that both patency capsule and radiologic studies showed equivalently high sensitivity and negative predictive value for the detection of significant intestinal strictures, defined as strictures that require surgery due to bowel obstruction or capsule retention [50]. Based on these reports, a recent guideline for VCE has recommended that significant small bowel strictures be ruled out before VCE by performing a patency capsule study and/or small bowel radiological study in suspected or established CD patients [18].

Third, the clinical impact of VCE on the management of suspected or established CD patients should be considered. Several retrospective studies suggested that VCE led to a definitive diagnosis of CD, changed the management of CD, or had a potential impact on the prognosis prediction of CD [51–56]. In a small prospective pediatric study, VCE reclassified 2 of 4 ulcerative colitis or indeterminate colitis patients and 8 of 10 suspected IBD patients as small bowel CD [57]. In another pediatric study, 50% of ulcerative colitis or unclassified IBD patients were finally diagnosed with CD and, more importantly, 94% of the suspected IBD patients were finally diagnosed as not having IBD [58]. The high negative predictive value (96%–100%) of VCE in other studies [22, 39, 59] suggests that VCE may have a significant advantage in the exclusion of the small bowel involvement of CD and IBD.

Given the risk of capsule retention, the absence of a standardized definition or description of VCE findings in small intestinal CD, and the lack of data related to the clinical impact or optimal indications of VCE, the role of VCE in the diagnosis of CD is still controversial and needs to be established more in detail. The second European evidence-based consensus on the diagnosis and management of CD stated that VCE can be used as a first-line test after exclusion of significant stenosis using a patency capsule or as a second-line test in patients in whom the clinical suspicion for CD remains high despite negative evaluations with ileocolonoscopy and radiologic examination [60]. The recent Korean guidelines for VCE in CD suggested that “in well-selected patients with a high suspicion of CD,” VCE is useful for diagnosing CD after negative ileocolonoscopy and small bowel radiologic examination [18]. Recently, the European Society of Gastrointestinal Endoscopy (ESGE) suggested different diagnostic recommendations for suspected CD and established CD [61]. In patients with suspected CD and negative ileocolonoscopy findings, the ESGE recommends VCE as the initial diagnostic modality for investigating the small bowel in the absence of obstructive symptoms or known stenosis [61]. On the other hand, in patients with established CD based on ileocolonoscopy findings, if the cross-sectional imaging tests were unremarkable or nondiagnostic and it is believed that VCE would influence patient management, the ESGE recommended VCE as a subsequent test [61].

### 3.2. Assessment of Disease Activity

Mucosal healing has been reported to be associated with better long-term prognosis in CD and has recently been considered to be a new target of CD treatment. VCE is useful to assess the disease activity and treatment response in small intestinal CD by providing information about the status of the small intestinal mucosa. Two VCE-based scoring systems have been suggested to quantify the extent of small intestinal disease in CD: the Lewis score and the Capsule Endoscopy Crohn's Disease Activity Index (CECDAI). The Lewis score uses three parameters—villous edema, ulceration, and stenosis—and the score for each parameter is weighted based on the extent and severity [62]. The whole small intestine observed by VCE is divided into three tertiles; the levels of villous edema and ulceration are scored for each tertile but stenosis is graded in the whole study, independent of the individual tertiles. When the scores were matched with the global assessment of mucosal disease activity, Lewis scores <135, 135–790, and >790 were categorized as normal, mild-to-moderate, and severe mucosal disease activity, respectively.

The other VCE-based CD disease activity assessment system, CECDAI [63], divides the small intestine into proximal and distal segments and uses different weights for three parameters: inflammation, extent of disease, and stricture. The final score is the sum of the proximal and distal segmental numbers and ranges from 0 (normal study) to 36 (severe disease). The interobserver agreement for the CECDAI score was excellent, with a Kappa value of 0.867. Good interobserver agreement of CECDAI was also validated in a prospective multicenter study of isolated small intestinal CD patients, but this study failed to show any significant correlation between CECDAI and the Crohn's Disease Activity Index (CDAI) or the Inflammatory Bowel Disease Quality of Life Questionnaire (IBDQ) [64]. In a retrospective study, the Lewis score showed a statistically significant correlation with CECDAI (*r* = 0.6324), but the Lewis score showed a better correlation with fecal calprotectin levels than CECDAI in CD patients with fecal calprotectin <100 *μ*g/g [65].

On the other hand, another retrospective multicenter study showed a poor correlation between fecal calprotectin and the Lewis score in CD patients, and exclusion of isolated colonic CD did not significantly improve their correlation. Therefore, the correlation between fecal calprotectin and the Lewis score or CECDAI is still controversial and should be investigated further. A recent retrospective study reported that therapeutic change was recommended in 14.5%, 48.1%, and 87.1% of patients with no small intestinal inflammation (Lewis score <135), mild small intestinal inflammation (Lewis score, 135–790), and moderate-to-severe small intestinal inflammation (Lewis score >790), respectively [55]. However, little is known about the roles of VCE-based disease activity indexes as indicators of treatment alterations or prognosis predictors. Further studies should be performed to determine the clinical implication of these indexes in the management of CD patients with small intestinal involvement.

### 3.3. Assessment of Postoperative Recurrence

Postoperative recurrence is quite common in CD and endoscopic recurrence usually precedes clinical recurrence. Therefore, postoperative surveillance of recurrence is an important issue in the management of CD. According to a prospective study that compared the usefulness of VCE and ileocolonoscopy as tools for monitoring postoperative CD recurrence, VCE seems not to be superior to ileocolonoscopy in the detection of endoscopic recurrence [25]. However, in that study, the investigators assessed the findings just around the anastomosis, which can be easily assessed by ileocolonoscopy in most postoperative cases. Another study suggested that VCE is more effective in the evaluation of recurrence after surgery for CD and is better tolerated than ileocolonoscopy [27]. Other studies also supported the usefulness of VCE as a noninvasive monitoring tool for postoperative recurrence of CD [26, 66], but further prospective studies with large number of patients are necessary to confirm the clinical usefulness of VCE for the detection of the postoperative recurrence of CD.

## 4. Conclusion

The diagnostic accuracy of VCE is superior to that of SBFT and CTE/CTEC and comparable to that of MRE in the diagnosis of CD. VCE is also more sensitive for the detection of subtle mucosal inflammation than MRE. Therefore, potential benefits from VCE can be anticipated in the diagnosis, management, and surveillance of postoperative recurrence CD. The therapeutic impact of VCE in CD could be derived from its accurate diagnosis and information on the extent of disease or disease activity, and several retrospective studies have reported that CD management changed after VCE in 53%–61.6% of patients [52, 54, 55]. VCE has a higher negative predictive value for the diagnosis of small intestinal CD than other modalities [22] and clinical and serologic disease activity indexes of CD show no or weak correlation with endoscopically assessed mucosal disease activities [64, 67, 68]. Therefore, even a normal VCE finding may be helpful for the management of symptomatic CD patients because it suggests a noninflammatory origin of symptoms such as those of irritable bowel syndrome. However, clinical experience with VCE for the diagnosis and management of CD is still limited despite the advantages of VCE over other modalities. The risk of capsule retention, lack of evidence on the clinical benefit, and lack of established indications for VCE in CD may limit the widespread use of VCE in clinical practice for suspected or established CD patients. Further studies are required to minimize the risk of capsule retention, to assess the clinical benefits and impact of VCE, and to establish the indications for VCE in CD.

## Figures and Tables

**Table 1:** Comparison of video capsule endoscopy with small bowel barium radiography: diagnostic yield or performance for suspected or established Crohn's disease.

Study	Design	Number of cases	Tests	Definition/description for positive findings	Diagnostic yield	Sensitivity/specificity
Eliakim et al. (2004) [12]	Prospective	35	VCE	Ulcers/erosions, erythema, aphthae, and nodular lymphoid hyperplasia	77%	NA
			SBFT	Wall thickening, nodularity in terminal ileum, and ulcers	23%	NA

Buchman et al. (2004) [11]	Prospective	30	VCE	Grade 0: normal Grade 1: erythema, isolated villi loss Grade 2: erosion, no ulcer Grade 3: ulcers, spontaneous bleeding, and/or stricture	70%	NA
			SBFT	Grade 0: normal Grade 1: minimal nodularity, ulcerations, normal luminal diameter, and <5 cm involvement Grade 2: more extensive ulcers, minimal luminal narrowing, and 5–10 cm involvement Grade 3: fistula, skip areas, extensive ulcerations, and >10 cm involvement	67%	NA

Dubcenco et al. (2005) [10]	Prospective	39	VCE	Presence of ≥3 ulcerations	67%	NA
			SBFT	Active inflammatory or fistulizing/perforating subtypes	21%	NA

Hara et al. (2006) [21]	Prospective	17	VCE	Erosions, ulcers, or strictures	71%	NA
			SBFT	Abnormal nodularity, loss of normal mucosal folds, linear ulcers, strictures, or fistulas	24%	NA

Efthymiou et al. (2009) [16]	Prospective	47 in total 29 established CD 26 suspected CD	VCE	Diffuse erythema, erosions, >3 aphthous ulcers, ulcers of different shape (serpiginous, linear, oval, and coalescing), and strictures	67%	NA
			EC	Fold thickening, aphthous ulceration, nodular appearances, ulcers at the mesenteric border, cobblestone appearance, fixed strictures, and fistulae	36%	NA

Solem et al. (2008) [23]	Prospective	27	VCE	NA^(1)^	NA	83%/53%
			EC	NA^(1)^	NA	65%/94%

Marmo et al. (2005) [14]	Prospective	31	VCE (*n* = 31)	Diffuse small bowel lesions, multiple (>3) erosions, or ulcers that were serpiginous, deep fissuring, coalescing, linear, or nodular	71% (22/31)	NA
			EC (*n* = 31)	Fold thickening, aphthous ulceration, granular appearances of the villi, nodular pattern, the presence of ulcerations on the mesenteric border, cobblestone appearance, fixed stenosis and/or strictures, and fistulae	26% (8/31)	NA

Chong et al. (2005) [15]	Prospective	22 established CD	VCE (*n* = 21)	Erosions/ulcers	77% (17/21)	NA
			EC (*n* = 21)	Narrowing or an irregular terminal ileum/neoterminal ileum	19% (4/21)	NA

Chong et al. (2005) [15]	Prospective	21 suspected CD	VCE (*n* = 21)	Erosions/ulcers	19% (4/21)	NA
			EC (*n* = 16)	NA	6% (1/16)	NA

Albert et al. (2005) [32]	Prospective	27 established CD	VCE (*n* = 14)	Aphthous mucosal lesions, irregularly shaped or fissural ulcers (occasionally associated with bleeding), cobblestone appearance, luminal narrowing due to oedema and/or fibrous scarring, and granularity with attenuated or lost vascular pattern	93% (13/14)	NA
			EC (*n* = 27)	NA	59% (16/27)	NA

Albert et al. (2005) [32]	Prospective	25 suspected CD	VCE (*n* = 13)	Same as above	92% (12/13)	NA
			EC (*n* = 14)	NA	29% (4/14)	NA

VCE, video capsule endoscopy; NA, not available; SBFT, small bowel follow-through; EC, enteroclysis; CD, Crohn's disease.

^(1)^No specific criteria for positive findings of CD were provided in the text. Diagnosis was made by a consensus panel of the coinvestigators in this study.

**Table 2:** Comparison of video capsule endoscopy with computed tomography enterography or enteroclysis: diagnostic yield or performance for suspected or established Crohn's disease.

Study	Design	Number of cases	Tests	Definition/description for positive findings	Diagnostic yield	Sensitivity/specificity
Voderholzer et al. (2005) [20]	Prospective	41 established CD	VCE	Small lesions (aphthoid ulcerations, villous denudation, and patchy erythema) and large lesions (such as cobblestone pattern, deep/fissural ulcerations)	61%	NA
			CTEC	Contrast enhancement of the mucosa and the other bowel wall layers, increased density of the peri-intestinal fat representing inflammatory changes and increased vascularity, separation of bowel loops, and possible lymphadenopathy The length and location of stenotic areas, the presence of fistulae, ulcerations, pseudodiverticulae, and polypous changes of the mucosa	29%	NA

Eliakim et al. (2004) [12]	Prospective	35 suspected CD	VCE	Ulcers/erosions, erythema, aphthae, and nodular lymphoid hyperplasia	77%	NA
			CTE	Wall thickening, nodularity in terminal ileum, and ulcers	20%	NA

Hara et al. (2006) [21]	Prospective	17 (8 suspected and 9 established CD)	VCE	Erosions, ulcers, or strictures	71%	NA
			CTE	Increased mucosal or wall enhancement, bowel wall thickening > 3 mm, fistulas, or abscesses	53%	NA

Solem et al. (2008) [23]	Prospective	27	VCE	NA^(1)^	NA	83%/53%
			CTE	NA^(1)^	NA	82%/89%

Jensen et al. (2011) [22]	Prospective	80	VCE^(2)^	More than 3 ulcerations (aphthous lesions or ulcers), irregular ulcers/fissures, or stenosis caused by fibrosis or inflammation	30%^(3)^	100%/91%
			CTE^(2)^	Mucosal ulcerations, bowel wall thickening, bowel wall hyperenhancement, small bowel stenosis, creeping fat, dilated vasa recta, and the presence of an abscess or fistula in conjunction to a diseased small bowel segment	33%^(3)^	76%/85%

CD, Crohn's disease; VCE, video capsule endoscopy; NA, not available; CTEC, computed tomography enteroclysis; CTE, computed tomography enterography.

^(1)^No specific criteria for positive findings of CD were provided in the text. Diagnosis was made by a consensus panel of the coinvestigators in this study.

^(2)^VCE and CTE were performed for 69 of 80 and 73 of 80 patients, respectively.

^(3)^Diagnostic yield for terminal ileal CD.

**Table 3:** Comparison of video capsule endoscopy with magnetic resonance enterography or enteroclysis: diagnostic yield or performance for suspected or established Crohn's disease.

Study	Design	Number of cases	Tests	Definition/description for positive findings	Diagnostic yield	Sensitivity/specificity
Albert et al. (2005) [32]	Prospective	25 suspected CD	VCE	Aphthous mucosal lesions, irregularly shaped or fissural ulcers (occasionally associated with bleeding), cobblestone appearance, luminal narrowing due to edema and/or fibrous scarring, and granularity with attenuated or lost vascular pattern	NA	92%/100%
			MRE	Thickening of the bowel wall (>4 mm) and enhancement of the bowel wall after application of intravenous contrast medium	NA	77%/80%
	Prospective	27 established CD	VCE	Same as above	93%	NA
			MRE	Same as above	88%	NA

Gölder et al. (2006) [33]	Prospective	18 (2 suspected and 16 established CD)	VCE	Grade 0: no inflammation Grade 1: <3 aphthous lesions or a single ulcer Grade 2: >3 aphthous lesions or >1 ulceration or an inflammatory stenosis	76%^(1)^	NA
			MREC	Bowel wall thickening with contrast enhancement, mesenteric injection, and enlarged lymph nodes	41%	NA

Tillack et al. (2008) [34]	Prospective	19 established CD	VCE	Grade 0: no mucosal pathology Grade 1: minor inflammation (focal denudation of villi, superficial aphthae and erosions, focal erythema, and <2 ulcers) Grade 2: major inflammation (>ulcers, deep ulcers, fissures, cobblestone pattern, and fibrinous exudates) Obstruction grade 0: no obstruction Obstruction grade 1: stenosis (delayed capsule propulsion, propagation stop)	95%	NA
			MRE	Grade 0: no mucosal or mural pathology Grade 1: minor inflammation (subtle irregularity of the fold pattern, subtly increased contrast uptake, no wall thickening, no submucosal edema, and no extramural hypervascularity) Grade 2: major inflammation (ulcers, deep mucosal fissures disrupting the fold pattern, cobblestone pattern, markedly increased contrast uptake, wall thickening > 4 mm, submucosal edema, extramural hypervascularity, and contrast enhancing lymphadenopathy (>15 mm)) Obstruction grade 0: no obstruction: stenosis with prestenotic dilation (luminal narrowing <5 mm)	95%	NA

Jensen et al. (2011) [22]	Prospective	80	VCE^(2)^	More than 3 ulcerations (aphthous lesions or ulcers), irregular ulcers/fissures, or stenosis caused by fibrosis or inflammation	30%^(3)^	100%/91%
			MRE^(2)^	Mucosal ulcerations, bowel wall thickening, bowel wall hyperenhancement, small bowel stenosis, creeping fat, dilated vasa recta, and the presence of an abscess or fistula in conjunction with a diseased small bowel segment	28%^(3)^	76%/85%

Wiarda et al. (2012) [24]	Prospective	38 (20 suspected and 18 established CD)	VCE^(4)^	Mild: erythematous and/or edematous mucosa and/or small ulcerative lesions (<0.5 mm) within otherwise normal appearing mucosa Moderate: larger ulcerative lesions (≥0.5 mm and <20 mm) Severe: large ulcerative lesions (≥20 mm) and/or significant stenotic lesions, with or without macroscopic signs of inflammation.	NA	57%/89% (for 25 nonstenotic CD)
			MREC	Bowel wall thickness >4 mm, intramural and mesenteric edema, mucosal hyperemia, wall enhancement and enhancement pattern and transmural ulcerations, and fistula formation	NA	73%/90% (for all participants)

CD, Crohn's disease; VCE, video capsule endoscopy; NA, not available; MRE, magnetic resonance enterography; MREC, magnetic resonance enteroclysis.

^(1)^Diagnostic yield for small intestinal CD.

^(2)^VCE and MRE were performed for 69 of 80 and 72 of 80 patients, respectively.

^(3)^Diagnostic yield for terminal ileal CD.

^(4)^VCE was not done for 13 patients showing small intestinal stenosis in MREC.

**Table 4:** Comparison of video capsule endoscopy with ileocolonoscopy or push enteroscopy: diagnostic yield or performance for suspected or established Crohn's disease.

Study	Design	Number of cases	Tests	Definition/description for positive findings	Diagnostic yield
Bloom et al. (2003) [29]	Prospective	16	VCE	NA	56%
			IL	NA	50%

Bourreille et al. (2006) [25]	Prospective	31 (for postoperative evaluation)	VCE	Erythema, villous denudation, erosion, and ulceration (Rutgeerts score ≥1 for the recurrence at the neoterminal ileum)	42–55% for neoterminal ileum, 66–72% for entire small bowel
			IL	Rutgeerts score ≥1	61%

Hara et al. (2006) [21]	Prospective	17 (8 suspected and 9 established CD)	VCE	Erosions, ulcers, or strictures	71%
			IL	Erosions, ulcers, or strictures	65%

Biancone et al. (2007) [26]	Prospective	17 (for postoperative evaluation)	VCE	Separate detection of ulcers, strictures, or stenosis in the neoterminal ileum and/or anastomosis	94%
			IL	Rutgeerts score ≥1	94%

Beltrán et al. (2007) [27]	Prospective	24 (for postoperative evaluation)	VCE	Aphthoid ulcerations, small ulcer, cobblestone pattern, and deep/fissural ulcerations	62% (15/21)
			IL	Aphthoid ulcerations, small ulcer, cobblestone pattern, and deep/fissural ulcerations	25% (6/21)

Chong et al. (2005) [15]	Prospective	22 established CD	VCE	Erosions/ulcers	77%
			PE	NA	14%

Chong et al. (2005) [15]	Prospective	21 suspected CD	VCE	Erosions/ulcers	19%
			PE	NA	0

CD, Crohn's disease; VCE, video capsule endoscopy; IL, ileocolonoscopy; PE, push enteroscopy.
